# Primary adrenal diffuse large B cell Lymphoma with Tumor thrombus in central adrenal vein: a case report and literature review

**DOI:** 10.1186/s12902-023-01496-y

**Published:** 2023-11-24

**Authors:** Qingfei Xing, Chunhai Hu, Qingqing Zhao, Chunling Zhang, Tingshuai Cao, Xinghua Gao, Li He, Feng Guo

**Affiliations:** 1 Department of Urology, Central Hospital Affiliated to Shandong First Medical University, Jinan, China; 2 Department of Medical Imaging, Central Hospital Affiliated to Shandong First Medical University, Jinan, China; 3 Department of pathology, Central Hospital Affiliated to Shandong First Medical University, Jinan, China; 4 Department of Health, Shandong Province Hospital, Jinan, China

**Keywords:** Primary adrenal Lymphoma (PAL), Tumor thrombus, Diffuse large B-cell Lymphoma(DLBCL), Cadrenal vein, Case report

## Abstract

**Background:**

Primary adrenal lymphoma (PAL) is a rare disease confined wholly or chiefly to extramural involvement. Tumor thrombus in the central adrenal vein, renal vein, and inferior vena cava has been reported in adrenal pheochromocytoma, adrenocortical carcinoma, adrenal metastasis carcinoma, and adrenal leiomyosarcoma. Primary adrenal diffuse large B cell lymphoma with tumor thrombus in the central adrenal vein has rarely been reported in the current study. ( We searched in PubMed, Web of Science databases, Embase, and Medline in the English language from 1970 to December 2022. The keywords used were “Primary adrenal lymphoma " and " tumor thrombus”.)

**Case presentation:**

In this report, we discuss the case of a 57-year-old woman who complained of abdominal discomfort following cold stimulation, low back pain, anorexia, fatigue, and weight loss for 1 year. Contrast-enhanced spiral computed tomography (CT) showed mild-to-moderate enhancement of the bilateral masses and central adrenal vein tumor thrombus. After an exhaustive study, the patient was diagnosed with primary adrenal diffuse large B-cell lymphoma. In the diagnosis of PAL, the possibility of a tumor embolism in the central adrenal vein, renal vein, or inferior vena cava should be considered, although this is rare.

## Background

Although the adrenal glands contain no lymphoid tissue, Primary adrenal lymphoma (PAL) is rare, accounting for < 1% of all non-Hodgkin’s lymphomas and 3% of primary extranodal lymphomas [1, 2]. The PAL is primarily bilateral. To date, approximately 250 cases have been described in the literature [3], but the majority of published articles on PAL were case reports or case series studies with only a limited number of patients. Previous studies have reported, adrenocortical pheochromocytoma, adrenocortical carcinoma, adrenal metastasis carcinoma,and adrenocortical leiomyosarcoma with tumor thrombi in the central adrenal vein, renal vein, and inferior vena cava. We report a case of primary adrenal diffuse large B-cell lymphoma with a tumor thrombus in the central adrenal vein, which is, to the best of our knowledge, reported for the first time.

## Case presentation

**Fig. 1:**
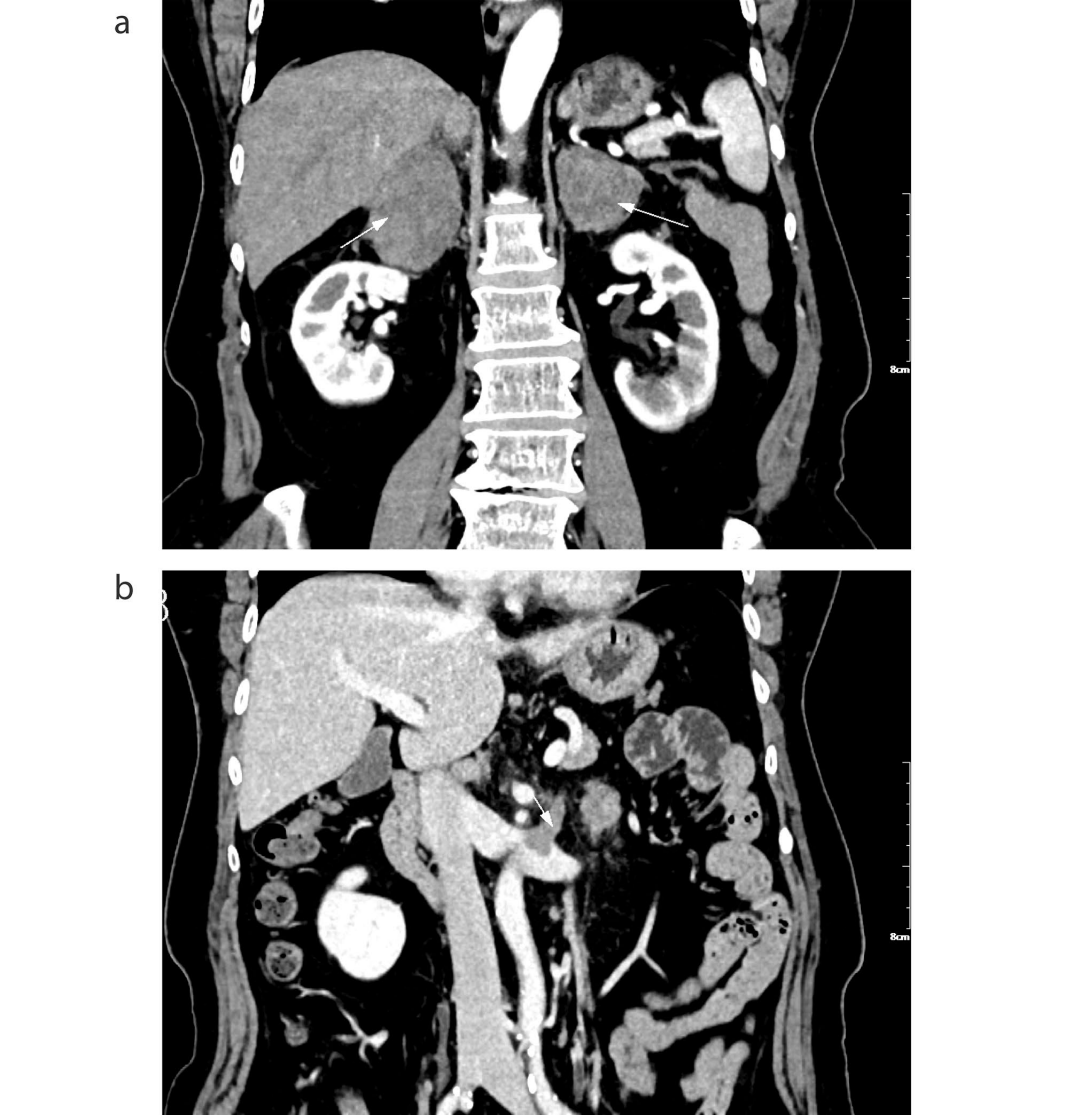
(**A**, **B**) Contrast-enhanced spiral computed tomography (CT) showed mild to moderate enhancement of bilateral masses and central adrenal vein tumor thrombus(arrow)

**Fig. 2:**
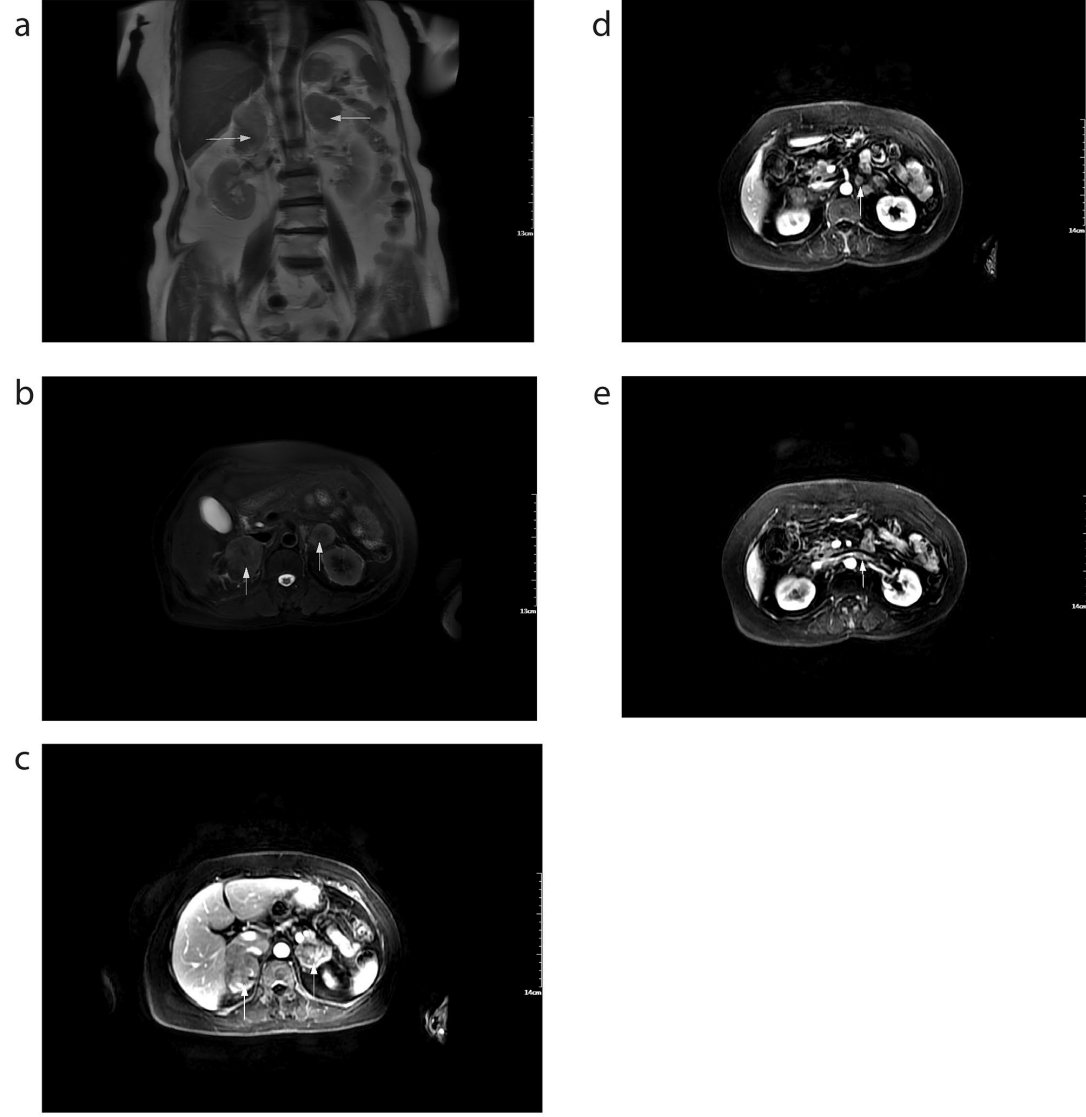
(**A**, **B**)Contrast-enhanced MR confirmed a long T1 and T2 signal shadow (arrow). Contrast-enhanced MRI revealed moderate enhancement in adrenal masses (**C**,arrow) and tumor thrombus in the central adrenal vein (**D**,arrow) and left renal vein (E,arrow)

A 57-year-old woman presented with complaints of abdominal discomfort following cold stimulation, lower back pain, anorexia, fatigue, and weight loss for 1 year. Recently, she underwent gastroscopy and colonoscopy,which were unremarkable, and subsequently underwent computed tomography (CT) of the abdomen. A CT scan revealed bilateral adrenal masses of hypodense tissue measuring 7 and 4 cm in diameter on the right and left sides, respectively. The patient was subsequently referred to the urology surgery clinic. Contrast-enhanced spiral computed tomography (CT) showed mild-to-moderate enhancement of the bilateral masses and central adrenal vein tumor thrombus (Fig. 1A,B). Contrast-enhanced MR confirmed a long T1 and T2 signal shadow (Fig. 2A,B). Contrast-enhanced MRI revealed moderate enhancement in the adrenal masses (Fig. 2C) and tumor thrombus in the central adrenal vein (Fig. 2D) and left renal vein (Fig. 2E).

Primary physical examination and basic laboratory tests, including aldosterone, cortisol, and catecholamines, were normal, and lactic dehydrogenase (LDH) levels were > 570 UI/L (120–250). Blood pressure was within normal limits, and no peripheral lymphadenopathy was detected on ultrasound. Primary adrenal insufficiency was diagnosed by a tetracosactide stimulation test, primary adrenal insufficiency was diagnosed. The patient received a 15 mg/day dose of hydrocortisone. Some symptoms, such as low back pain, anorexia, and fatigue were relieved.

**Fig. 3:**
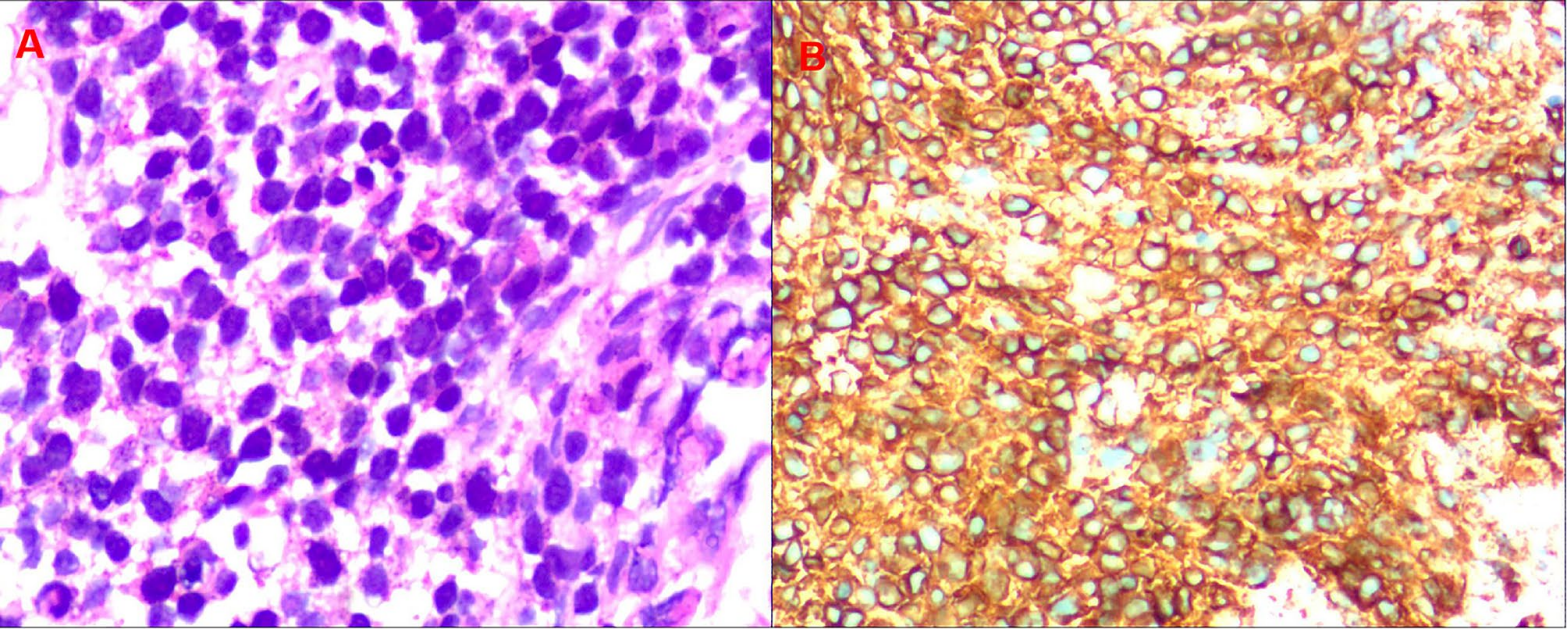
(**A**) Hematoxylin and eosin staining showed nuclear pleomorphism. (**B**) Immunohistochemical staining for the B-cell marker CD20 was positive

A biopsy guided by a CT scan was performed for diagnostic purposes. The microscopic examination showed tumor composed of small and intermediate cells with pleomorphic nuclei and a high mitotic figures (Fig. 3A). On immunohistochemistry, the tumor cells were diffusely positive for B cell markers CD20 (Fig. 3B), and negative for CD21, CD3, CD34, CD5, Cytokeratin, Vimentin, CD21, CA9, CgA, CR and inhibin. Ki67 proliferating index was 70% in the tumor cells. Overall, the histopathological analysis revealed diffuse large B-cell lymphoma (DLBCL).

The patient refused additional treatment including chemotherapy for personal reasons.

**Fig. 4:**
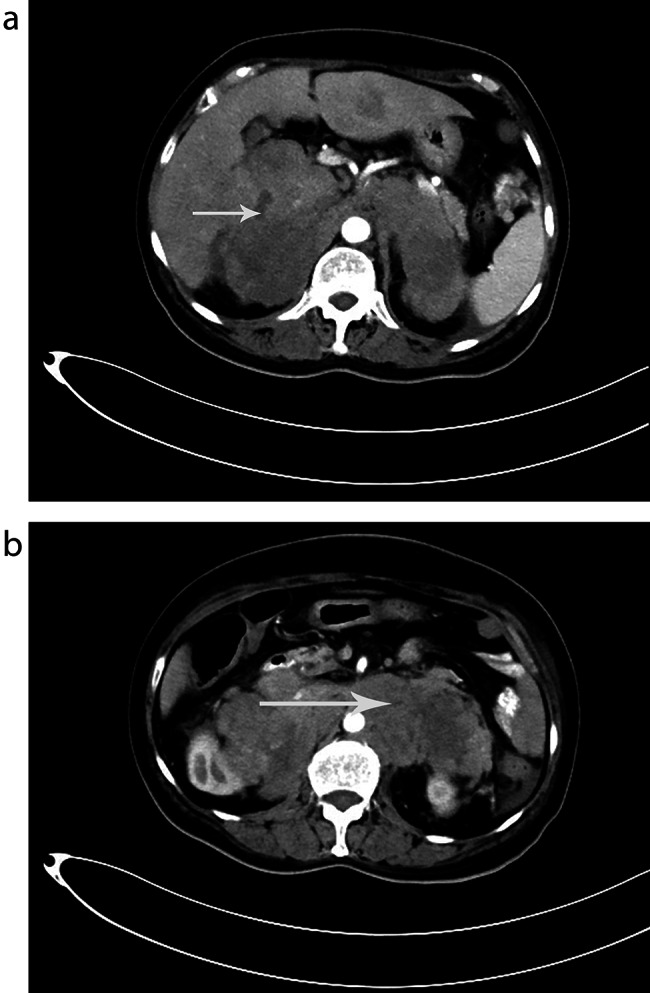
CT revealed liver(**A**,arrow) and retroperitoneal lymph node metastases(**B**,arrow)

This CT scan, performed 30 days after the first CT scan, showed a rapid progression of the disease with additional enlargement of the adrenal mass. This patient underwent a CT scan after needle biopsy of the adrenal mass for approximately 3 weeks, which revealed liver (Fig. 4A) and retroperitoneal lymph node metastases (Fig. 4B). The patient died of acute myocardial infarction before chemotherapy.

## Discussion

PAL is extremely rare, with approximately 250 cases in the medical literature, and the most common PAL is non-Hodgkin’s lymphoma [3]. Most primary adrenal DLBCL (PA-DLBCL) is of a non-germinal center B cell (non-GCB) phenotype. PAL typically occurs in elderly and male, with a median age at presentation ranging from 48 to 68 years and a male/female ratio of 1.8:1–7:1 [3–7]. Simultaneous involvement of the bilateral adrenal glands is common, and bilateral PAL accounts for 75% of PAL cases [3].

However, the exact pathogenesis of PAL remains unclear. Epstein-Barr virus infection, autoimmune-associated infections, immune dysfunction, and mutations in p53 and c-kit genes, which have been implicated in pathogenesis, have been described in previous studies [2, 8, 9].

PAL usually has no excretory endocrine function and the symptoms are due to the pressure effect of the mass [3], whereas adrenal insufficiency usually exists.The most common manifestations were B symptoms, which include unexplained fever, weight loss, night sweats (68%), vague abdominal pain (42%), and fatigue (36%) [3], some of which were present in the current patient.There is no correlation between tumor size and adrenal insufficiency [10]. Generally, obvious clinical manifestations of adrenal insufficiency tend to appear when > 90% of the adrenal gland is damaged [11]. It can improve with the destruction of the lymphomatous tissue at the end of the chemotherapy cycle. The incidence of adrenal insufficiency in PAL was 61% [3]. Compared with most metastatic cancers other than lymphoma, the high incidence of adrenal insufficiency in patients with PAL is primarily related to the functional and, cytokine-driven paracrine effects of lymphoma cells in the adrenal microenvironment. However, metastatic carcinoma is mainlycaused by overgrowth that eventually compresses or replaces the normally functioning adrenal parenchyma, or impairs the local vascular supply to the gland, leading to intralesional or parenchymal hemorrhage, necrosis, or infarction. Reports on the incidence of adrenal insufficiency in adrenal metastases are few and old, making comparative analyses of PAL highly problematic [12, 13].Further research is needed to determine the exact mechanism of adrenal insufficiency in PAL. In cases of bilateral adrenal involvement, adrenal insufficiency should be screened using the tetracosactide stimulation test. It is essential to be mindful of this, as it can save some time and relieve some symptoms, as in this case.

Diagnostic imaging included CT, MRI and PET-CT. CT can be used to evaluate adrenal masses for localization, visualization, and characterization. PAL is a metabolically hyperactive, hypovascular tumor presenting as a predominantly low-density and slight-to-moderate enhancement on CT [3]. In this case, the median CT value of PAL was 37 HU, and contrast-enhanced CT revealed a “slight to moderate enhancement” pattern, which is different from adrenocortical carcinoma and pheochromocytoma, which are hypervascular lesions that show significant enhancement. Contrast-enhanced CT revealed a tumor embolism in the central adrenal vein. Contrast-enhanced MRI was performed to demonstrate a tumor embolism in the central adrenal vein. Adrenocortical carcinoma was suspected due to a tumor embolism in the central adrenal vein, and surgery was considered. However, PAL cannot be ruled out radiologically. A needle core biopsy, guided by a CT scan, was performed to obtain a definitive diagnosis. Given the large surgical trauma, the patient chose needle biopsy to confirm the diagnosis. Following percutaneous biopsy of the left adrenal gland, histopathological examination revealed the presence of DLBCL. FDG-PET seems to be an ideal test, especially in evaluating treatment response to multimodal treatment, because FDG is only taken up by residual tumors and not by necrotic tumors or fibrosis [14].

Treatments for PA-DLBCL include surgery, combination chemotherapy, and surgery followed by chemotherapy and/or radiation therapy. Surgery is mainly used to diagnose PAL, but the status of surgery has been significantly reduced owing to the increased diagnostic success rate of interventional puncture. The recommended treatment regimen for PAL is rituximab, cyclophosphamide, doxorubicin, vincristine, and prednisone (R-CHOP), a classical chemotherapy regimen, with chemotherapy being the typical treatment, which increases the overall survival of patients with DLBCL [15]. Research suggested that the autologous stem cell transplant may further increase overall survival [16].

This disease appears to have a poor prognosis, with a median survival of less than one year [17]. According to previous reports, advanced stage, large tumor size, presentation with adrenal insufficiency, bilateral adrenal involvement, and high LDH levels are related to poor prognosis in PAL [15, 18–20]. This CT scan, performed 30 days after the first CT scan, showed rapid progression of the disease, with additional enlargement of the adrenal masses. The patient underwent a CT scan approximately 3 weeks after needle biopsy of the adrenal mass, which revealed metastases in the liver and retroperitoneal lymph nodes. The patient died of acute myocardial infarction before chemotherapy.

The diagnosis of bilateral adrenal tumors, like that of unilateral adrenal tumors, includes both localization and qualitative diagnosis.However, with the development of imaging techniques, the diagnosis of bilateral adrenal tumors has become easy, and qualitative diagnosis plays an important role in guiding the treatment of adrenal tumors.

Bilateral adrenal tumors can be observed in primary or secondary adrenal lymphoma, adrenal cortical carcinoma, pheochromocytoma, adrenal metastases, and bilateral adrenal macronodular hyperplasia.

PAL is a histologically proven lymphoma involving one or two adrenal glands with two characteristics: no prior history of lymphoma in other sites; if lymph nodes or other organs are involved, the adrenal gland is the main lesion site, and adrenal lymphoma:presentation, management, and prognosis). Owing to the lack of specific clinical manifestations of PAL, the diagnosis should be combined with auxiliary examination. B-ultrasonography, abdominal CT, MRI, PET-CT, bone marrow puncture, immunohistochemical staining, and chromosome karyotype analysis are of great significance in assisting the diagnosis of PAL. The diagnosis depends on pathology and ultrasound-guided adrenal needle biopsy.

Patients with adrenal metastatic carcinoma have a history of primary tumors, including lung cancer, breast cancer, kidney cancer, melanoma, thyroid cancer, and colon cancer.

Pheochromocytoma originates in the adrenal medulla. Most pheochromocytoma secrete catecholamines and cause corresponding clinical symptoms. More than 85% of patients have persistent or paroxysmal hypertension and a series of other metabolic disorders, and most patients have elevated plasma and urine concentrations of catecholamines and their metabolites.

Adrenocortical carcinoma presents as an irregular, lobulated mass with uneven density, blurred edges, and ambiguous boundaries, and signs of invasion.

Bilateral adrenal macronodular hyperplasia is a rare adrenal hyperplasia of unknown etiology. Owing to different levels of cortisol secretion, there may be different clinical manifestations such as Cushing’s syndrome and subclinical Cushing’s syndrome. CT revealed bilateral adrenal nodular hyperplasia.

Pheochromo cytoma, adrenal cortical carcinoma, and adrenal metastatic tumors have been reported with renal vein and inferior vena cava tumor thrombus, while cases of PAL with renal vein and inferior vena cava tumor thrombus have not been reported in China [21]. According to our investigation, the progressive tumor embolism in the central adrenal vein caused by PAL is the first reported case in China and the second case in the world.

## Conclusion

In conclusion, because it is a rare disease, additional research on PAL is needed to enhance the management of this lesion, explore the mechanisms of tumor embolism formation, and attract the attention of clinicians. PAL with poor prognosis must be diagnosed at an early stage to improve survival. In the diagnosis of PAL, the possibility of a tumor embolism in the central adrenal vein, renal vein, or inferior vena cava should be considered, although this is rare.

## Data Availability

The datasets used and/or analyzed during the current study are available from the corresponding author upon reasonable request.

## Abbreviations

PAL: Primary adrenal lymphoma
DLBCL: Diffuse large B-cell lymphoma
CT: Computed tomography scan
LDH: Lactic dehydrogenase
MRI: Magnetic resonance imaging
PA-DLBCL: Primary adrenal DLBCL
